# Bacterial Symbionts Confer Thermal Tolerance to Cereal Aphids *Rhopalosiphum padi* and *Sitobion avenae*

**DOI:** 10.3390/insects13030231

**Published:** 2022-02-25

**Authors:** Muhammad Zeeshan Majeed, Samy Sayed, Zhang Bo, Ahmed Raza, Chun-Sen Ma

**Affiliations:** 1 State Key Laboratory for Biology of Plant Diseases and Insect Pests, Climate Change Biology Research Group, Institute of Plant Protection, Chinese Academy of Agricultural Sciences, Beijing 100193, China; zhangbo05@caas.cn; 2 Department of Entomology, College of Agriculture, University of Sargodha, Sargodha 40100, Pakistan; 3 Department of Science and Technology, University College-Ranyah, Taif University, B.O. Box 11099, Taif 21944, Saudi Arabia; s.sayed@tu.edu.sa; 4 State Key Laboratory for Biology of Plant Diseases and Insect Pests, Cereal Fungal Diseases Research Group, Institute of Plant Protection, Chinese Academy of Agricultural Sciences, Beijing 100193, China; ahmed_scuafdo@yahoo.com; 5 Department of Plant Pathology, Sub-Campus Depalpur, University of Agriculture, Okara 56300, Pakistan

**Keywords:** wheat aphids, thermal traits, critical thermal maxima, chronic temperature tolerance, aphid endosymbionts, bacterial gene abundance

## Abstract

**Simple Summary:**

This study assesses the putative association between the chronic and acute thermal tolerance of cereal aphids *Rhopalosiphum padi* (L.) and *Sitobion avenae* (F.) and the abundance of their bacterial symbionts. Thermal tolerance indices were determined for 5-day-old apterous aphid individuals and were associated with the aphid-specific and total bacterial symbionts’ gene abundance (copy numbers). The results show a significantly higher bacterial symbionts’ gene abundance in temperature-tolerant aphid individuals than the susceptible ones for both aphid species. Moreover, the gene abundance of total (16S rRNA) bacteria and most of the aphid-specific bacterial symbionts for both cereal aphid species were significantly and positively associated with their critical thermal maxima values. Overall, the findings of the study suggest the potential role of the bacterial symbionts of aphids in conferring thermal tolerance to their hosts.

**Abstract:**

High-temperature events are evidenced to exert significant influence on the population performance and thermal biology of insects, such as aphids. However, it is not yet clear whether the bacterial symbionts of insects mediate the thermal tolerance traits of their hosts. This study is intended to assess the putative association among the chronic and acute thermal tolerance of two cereal aphid species, *Rhopalosiphum padi* (L.) and *Sitobion avenae* (F.), and the abundance of their bacterial symbionts. The clones of aphids were collected randomly from different fields of wheat crops and were maintained under laboratory conditions. Basal and acclimated CTmax and chronic thermal tolerance indices were measured for 5-day-old apterous aphid individuals and the abundance (gene copy numbers) of aphid-specific and total (16S rRNA) bacterial symbionts were determined using real-time RT-qPCR. The results reveal that *R. padi* individuals were more temperature tolerant under chronic exposure to 31 °C and also exhibited about 1.0 °C higher acclimated and basal CTmax values than those of *S. avenae*. Moreover, a significantly higher bacterial symbionts’ gene abundance was recorded in temperature-tolerant aphid individuals than the susceptible ones for both aphid species. Although total bacterial (16S rRNA) abundance per aphid was higher in *S. avenae* than *R. padi*, the gene abundance of aphid-specific bacterial symbionts was nearly alike for both of the aphid species. Nevertheless, basal and acclimated CTmax values were positively and significantly associated with the gene abundance of total symbiont density, *Buchnera aphidicola*, *Serratia symbiotica*, *Hamilton defensa*, *Regiella insecticola* and *Spiroplasma* spp. for *R. padi*, and with the total symbiont density, total bacteria (16S rRNA) and with all aphid-specific bacterial symbionts (except *Spiroplasma* spp.) for *S. avenae*. The overall study results corroborate the potential role of the bacterial symbionts of aphids in conferring thermal tolerance to their hosts.

## 1. Introduction

Global warming is primarily manifested not only by a gradual rise in the Earth’s average temperature, but also by the occurrence of frequent extreme high temperature events. Such high temperature events can influence population performances and the demographic parameters of insects, both in temporal and spatial scales [1,2,3].

Inferring the influence of extreme climate events on insects necessitates a better comprehension of their thermal tolerance limits and the mechanisms behind it [4,5,6]. Previous works have shown the significant impact of extreme high temperatures, both under acclimated and chronic exposures, on the physiology and thermal biology of invertebrates, including aphids [1,7,8,9,10,11,12,13]. Some studies, for instance, have demonstrated that different aphid species respond differently to chronic and acclimated temperature exposures [3,9,10,11,12,13]. However, the underlying mechanisms for such thermal impacts on aphids’ biology and ecology are largely unknown.

Aphids have been model systems to study insect–microbial symbiont interactions. They harbor many symbiotic bacteria within their bodies, including primary or obligate (*Buchnera aphidicola*) and secondary or facultative (*Serratia symbiotica*, *Hamiltonella defensa*, *Regiella insecticola*, *Rickettsia* spp. and *Spiroplasma* spp.) symbionts [14,15,16,17,18]. These aphid-specific symbiotic bacteria perform various functions, such as obligatory *B. aphidicola* provides nutritional supplementation and other facultative endosymbionts confer their hosts resistance to natural enemies and environmental extremities [19,20,21,22,23,24]. Previous studies have demonstrated the potential mediation of heat tolerance in aphids by their obligate and facultative bacterial symbionts [25,26,27,28].

In this paper, we address the putative effects of high temperature exposure on the thermal tolerance of aphids and their bacterial symbionts’ abundance. We intend to understand if short-term heat acclimation and chronic exposure to high temperatures would mediate any effect on the thermal tolerance, survival and abundance of symbiotic bacterial of aphids and if the thermal tolerance traits of aphids correlate to the abundance of their respective bacterial symbionts. To this end, individuals of two cereal aphids, i.e., bird cherry-oat aphid, *Rhopalosiphum padi* (L.), and English grain aphid, *Sitobion avenae* (F.), were chronically exposed to 31 °C (until death) and were acclimated to 34 °C for 3 h, before subjecting them to basal and acclimated critical thermal maxima (CTmax) determination. The abundance of aphid-specific bacterial endosymbionts was assessed using qPCR and their correlation with host thermal traits was worked out.

## 2. Materials and Methods

### 2.1. Collection and Rearing of Aphids

In this study, cereal aphids *R. padi* and *S. avenae* were studied as model species because of their differential performance under extreme temperature regimes [1,3]. About 100 wild clones of *R. padi* and *S. avenae* were randomly collected from winter wheat (*Triticum aestivum* L.) fields near the Henan (35°59′26.0″ N 114°31′37.9″ E) and Hebei (39°30′36.4″ N 115°55′58.2″ E) provinces of China. These aphid clones were transferred separately under cool conditions in plastic tubes. These clones were reared separately on wheat seedlings. Rearing was conducted up to F_3_ generations under standard conditions, i.e., at 65 ± 5% relative humidity, 22 ± 1 °C temperature and under 16 h: 8 h light:dark photoperiod.

### 2.2. Experiment of Chronic Thermal Exposure

**Figure 1:**
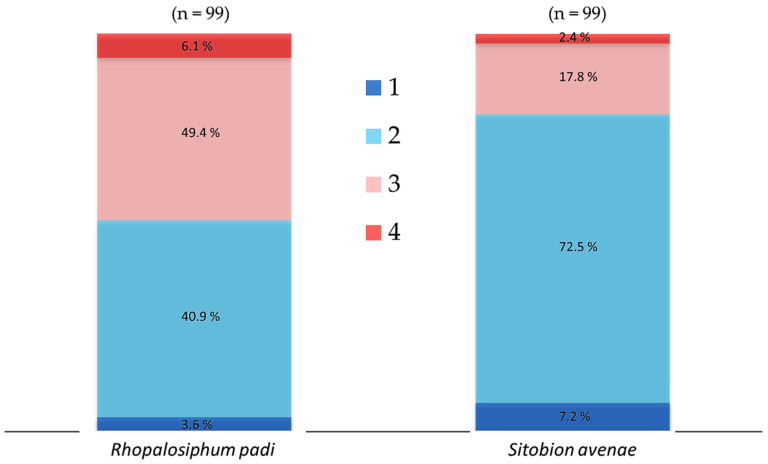
Cumulative percent mortality of cereal aphids *Rhopalosiphum padi* and *Sitobion avenae* exposed chronically to 31 °C. For each species, 33 aphid individuals were exposed from each of the laboratory-reared F_1_, F_2_ and F_3_ generations. Dead aphid individuals were divided into four mortality time periods, i.e., 1–4, representing susceptible-to-tolerant thermal threshold levels. For *R. padi*, the mortality time periods 1–4 refer to the individuals that died within 6–24, 24–48, 48–72 and 72–96 h, respectively, while for *S. avenae*, mortality time periods 1–4 refer to the individuals died within 6–18, 18–36, 3–54 and 54–66 h, respectively.

In order to determine the association between chronic heat tolerance and the gene abundance of aphid-specific bacterial symbionts, we collected three batches of aphids from the three generations reared in the laboratory. There were 33 apterous (5 days old) active and healthy aphid individuals in each batch. The tested aphids were reared individually on wheat leaves plugged in a moist sponge fixed in plastic tubes (30 mm diameter and 100 mm length) and the leaves were changed on alternate days. The three batches of aphids were exposed until death to 31 °C in a climate chamber (RXZ-280B; Jiangnan Ltd., Ningbo, China) set at 55–70% relative humidity and under 16 h: 8 h light:dark photoperiod. The observations were made at regular intervals of 3–6 h until the end of experiment and dead aphids were transferred immediately in vials containing 95% ethanol and were preserved at −20 °C in a freezer for the extraction of DNA. For the comparison of aphid-specific and total bacterial symbiont communities, dead aphid individuals were categorized into four mortality time periods as per their tolerance to chronic temperature (31 °C). For *R. padi*, mortality time period 1–4 refer to the individuals that died within 6–24, 24–48, 48–72 and 72–96 h, respectively, while for *S. avenae*, mortality time period 1–4 refer to the individuals that died within 6–18, 18–36, 3–54 and 54–66 h, respectively (Figure 1).

### 2.3. Basal and Acclimated Critical Thermal Maxima Determination

To determine basal critical thermal maxima (CTmax), we collected 3 batches of aphids (each batch with 33 individuals) from the 3 generations of aphids reared in laboratory. The tested aphids were placed individually in a multi-well transparent plastic arena, which was then hanged in a vertical position in the middle of a double-layered glass container (20 × 30 cm) of a programed glycol bath with an accuracy of ± 0.01 °C (Ministat 230-cc-NR; Huber Ltd., Berching, Germany). The temperature in the container was first maintained at 21 °C for 20 min and then was augmented gradually by 0.1 °C min^−1^ until the death of all test aphid individuals. Panasonic HDC-HS700 HD Camcorder (Panasonic, Osaka, Japan) was employed for recording the entire behavior of aphids in the arena plate during whole heating process. The temperature at which an aphid individual showed body spasms and lost its ability to move was noted as its CTmax value [29]. At the end of experiments, dead aphids were transferred immediately in vials containing 95% ethanol and were preserved at −20 °C in a freezer for the extraction of DNA.

To determine acclimated CTmax, we collected 3 batches of aphids (each batch with 33 individuals) from the 3 generations of aphids reared in laboratory. The tested aphids were first acclimated at 34 °C for 3 h (this acclimation duration was selected as aphids did not lose their fitness up to 3 h in a pilot test; see Figure S1), and then were subjected to acclimated CTmax determination using same protocol as mentioned above for basal CTmax.

### 2.4. Quantification of Aphid Bacterial Symbionts

After surface sterilization with 1.0% sodium hypochlorite, the total genomic DNA was extracted from the preserved aphid individuals using TIANamp^®^ genomic DNA Kit (Tiangen Biotech, Beijing, China), according to the protocol provided by the manufacturer. Purified DNA was eluted from each sample using 200 µL of Tris-EDTA buffer provided in the DNA extraction kit, and after quantification by NanoDrop^TM^ spectrophotometer (ND-1000, Thermo Fisher Scientific, Waltham, MA, U.S.A.), it was preserved at -20°C in a freezer until downstream molecular analysis. Diagnostic PCR tests were carried out with MyCycler^TM^ thermal cycler (Bio-Rad Laboratories Inc., Hercules, CA, U.S.A.) for the detection and subsequent optimization of annealing temperatures of primers. Primer pairs along with their sequences used for the diagnostic PCR amplifications of obligate and facultative bacterial symbionts of aphid individuals are detailed in Table 1.

For RT-qPCR amplifications, linearized recombinant plasmids (pGEM-T Easy Vector; Promega) were prepared using pGM-T Cloning Kit (Tiangen Biotech, Beijing, China) having a standard sequence inserts of target genes. Standard curves were created using serial dilutions (10-fold) of purified linearized plasmids containing 10^1^ to 10^9^ copies of the targeted bacterial genes. Each RT-qPCR reaction mixture of 20 µL was constituted of 10 μL 2× SuperReal PreMix Plus (Tiangen, Beijing, China), 0.6 μL of each of the 10μM forward and reverse primers, 8.4 μL of ddH_2_O and 1 μL of 10 ng μL^−1^ of DNA template. The thermal protocol used for RT-qPCR amplifications included an enzyme activation step of 94 °C for 600 s, 40 cycles with denaturation, annealing and extension steps, respectively at 94 °C for 30 s, 55 °C for 30 s and 72 °C for 60 s. For each sample, three independent biological and technical replicates were run.

### 2.5. Statistical Analysis

Data were statistically analyzed using Statistix V. 8.1^®^ (Tallahassee, FL, USA) analytical software. For each aphid species, the comparison of basal and acclimated CTmax indices was conducted by Student’s paired *t*-test at *p* ≤ 0.05. Similarly, Student’s paired *t*-test was employed to compare the gene abundance of aphid-specific bacterial symbionts between different critical thermal maxima (CTmax) treatments or between both aphid species. Spearman’s rank correlation analysis was worked out to explore the potential association among the gene abundance of aphid-specific endosymbionts and thermal indices of aphids.

## 3. Results

### 3.1. Symbionts Gene Abundance and Chronic Thermal Tolerance

When *R. padi* were exposed to 31 °C, mortality was less than 5% within 36 h, then increased rapidly and reached a high level of 25% between 36 to 66 h, and then maintained a low level of less than 10% between 66–96 h (Figure S2). The proportion of tested *R. padi* individuals that died within the mortality time periods 1–4 when exposed chronically to temperature of 31 °C was 3.6, 49.4, 40.9 and 6.1%, respectively (Figure 1). For *S. avenae*, when the tested aphids were exposed to 31 °C, mortality was less than 10% within 18 h, then it increased dramatically and reached a peak of ca. 40% between 18 to 36 h, and then decreased gradually from 15% to very low levels between 36–66 h (Figure S2). The tested *S. avenae* individuals that died within the mortality time periods 1–4 when exposed chronically to temperature of 31 °C were 7.2, 72.5, 17.8 and 2.4%, respectively (Figure 1).

**Figure 2:**
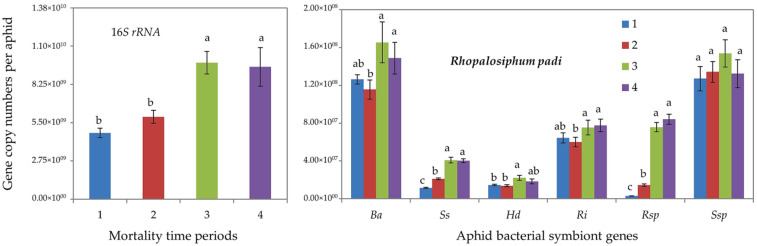
Abundance (mean gene copy numbers ± SD; *n* = 20) of bacterial symbionts of aphids (*Rhopalosiphum padi*) under chronic exposure to 31 °C. Dead aphid individuals were divided into four mortality time periods, i.e., 1–4, representing susceptible-to-tolerant thermal threshold levels. 16S rRNA = total eubacterial rDNA gene; Ba = *Buchnera aphidicola*; Ss = *Serratia symbiotica*; Hd = *Hamiltonella defensa*; Ri = *Regiella insecticola*; Rsp = *Rickettsia* spp.; Ssp = *Spiroplasma* spp. Different letters at the tops of the treatment bar show significant differences between treatments (one-way ANOVA; *p* ≤ 0.05).

**Figure 3:**
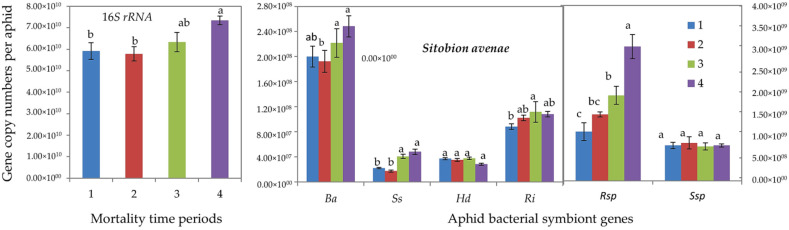
Abundance (mean gene copy numbers ± SD; *n* = 20) of bacterial symbionts of aphids (*Sitobion avenae*) under chronic exposure to 31 °C. Dead aphid individuals were divided into four mortality time periods, i.e., 1–4, representing susceptible-to-tolerant thermal threshold levels. 16S rRNA = total eubacterial rDNA gene; Ba = *Buchnera aphidicola*; Ss = *Serratia symbiotica*; Hd = *Hamiltonella defensa*; Ri = *Regiella insecticola*; Rsp = *Rickettsia* spp.; Ssp = *Spiroplasma* spp. Different letters at the tops of the treatment bar show significant differences between treatments (one-way ANOVA; *p* ≤ 0.05).

Furthermore, qPCR determinations showed that tolerant *R. padi* individuals (of mortality time periods 3 and 4) harbored significantly higher gene copy numbers of total (16S rRNA) bacterial symbionts and of all aphid-specific bacterial symbionts, i.e., *B. aphidicola*, *S. symbiotica*, *H. defensa*, *R. insecticola* and *Rickettsia* spp. (Figure 2). For *S. avenae*, the difference was significant only for 16S rRNA, *S. symbiotica* and *Rickettsia* spp. (Figure 3).

### 3.2. Symbionts Gene Abundance and Acute Thermal Tolerance

**Figure 4:**
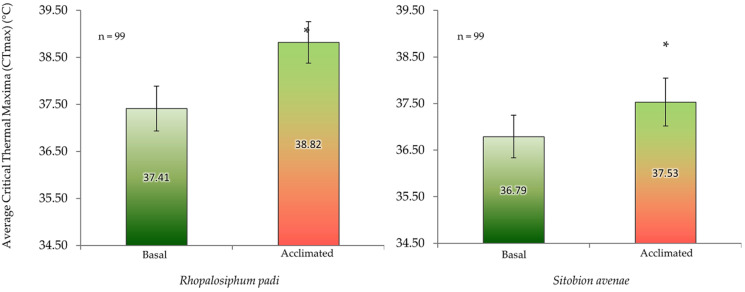
Basal and acclimated critical thermal maxima (CTmax) (mean ± SD; *n* = 99) of cereal aphids *Rhopalosiphum padi* and *Sitobion avenae*. Asterisks indicate significant difference among acclimated and basal CTmax for each aphid species (Student’s paired *t*-test; *p* ≤ 0.05).

Mean acclimated and basal CTmax values were 38.82 ± 0.44 °C and 37.41 ± 0.48 °C for *R. padi* and 37.53 ± 0.51 °C and 36.79 ± 0.46 °C for *S. avenae*(Figure 4). Acclimated individuals of both aphid species exhibited significantly higher CTmax values than the non-acclimated aphids (basal CTmax) (Figure 4). On average, CTmax values of *S. avenae* were about 1.0 °C less than those of *R. padi*.

**Figure 5:**
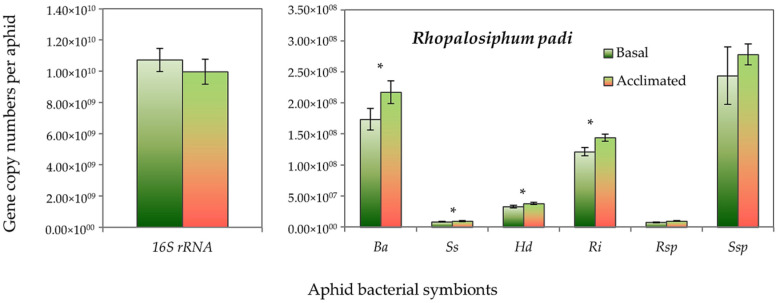
Abundance (mean gene copy numbers ± SD; *n* = 99) of bacterial symbionts of *Rhopalosiphum padi* aphids in acclimated and basal treatments. 16S rRNA = total eubacterial *r*DNA gene; Ba = *Buchnera aphidicola*; Ss = *Serratia symbiotica*; Hd = *Hamiltonella defensa*; Ri = *Regiella insecticola*; Rsp = *Rickettsia* spp.; Ssp = *Spiroplasma* spp. Asterisks signify significant difference between acclimated and basal treatments (Student’s paired *t*-test; *p* ≤ 0.05).

**Figure 6:**
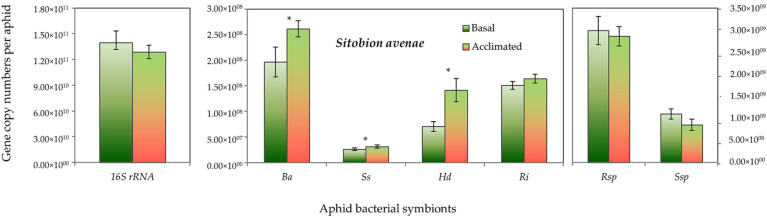
Abundance (mean gene copy numbers ± SD; *n* = 99) of bacterial symbionts of *Sitobion avenae* aphids in acclimated and basal treatments. 16S rRNA = total eubacterial *r*DNA gene; Ba = *Buchnera aphidicola*; Ss = *Serratia symbiotica*; Hd = *Hamiltonella defensa*; Ri = *Regiella insecticola*; Rsp = *Rickettsia* spp.; Ssp = *Spiroplasma* spp. Asterisks signify significant difference between acclimated and basal treatments (Student’s paired *t*-test; *p* ≤ 0.05).

Both aphid species had higher absolute gene copy numbers of total bacteria (16S rRNA) in non-acclimated (basal) aphids than the acclimated ones, but it did not reach the significant level. However, the gene copy numbers of *B. aphidicola*, *S. symbiotica*, *H. defensa* and *R. insecticola* in *R. padi* were significantly higher in the acclimated than in the non-acclimated (basal) aphid individuals (Figure 5). Likewise, in *S. avenae*, the gene abundance of *B. aphidicola*, *S. symbiotica* and *H. defensa* were moderately, but significantly, higher in the acclimated than non-acclimated (basal) aphid individuals (Figure 6). The mean gene abundance of total bacterial (16S rRNA) and aphid-specific bacterial symbionts was higher in *S. avenae* than *R. padi*, except the abundances of B. aphidicola and R. insecticola gene copy numbers, which were similar in both species (Figure S3).

Nevertheless, a significant rank correlation was recorded between the thermal tolerance indices and the abundance (gene copy numbers) of total symbiont density, *B. aphidicola*, *S. symbiotica*, *H. defensa*, *R. insecticola* and *Spiroplasma* spp. for *R. pad*i, and with the total symbiont density, total bacteria (16S rRNA) and with all aphid-specific bacterial symbionts (except *Spiroplasma* spp.) for *S. avenae* (Table 2).

## 4. Discussion

Extreme high temperature events exert significant effects on the thermal tolerance of aphids as simulated through chronic and acclimated exposures to high temperatures [7,9,10,11,12,13]. However, do the bacterial symbionts of aphids mediate the chronic and acute thermal tolerance of their hosts? This question remains to be cleared. This study was performed to determine the association between the chronic and acute thermal tolerance of *R. padi* and *S. avenae* aphids, and the gene abundance of their total (16S rRNA), secondary or facultative (*S. symbiotica*, *H. defensa, R. insecticola*, *Rickettsia* spp. and *Spiroplasma* spp.) and primary or obligate (*B. aphidicola*) endosymbiotic bacteria [17,18].

The findings of this study corroborated the fact that the *R. padi* species is more heat tolerant and exhibits higher evolutionary potential to high temperature events as compared to *S. avenae* [10,13,30]. Moreover, the acclimation-induced enhanced thermal threshold observed in both aphid species is in line with previous studies demonstrating the greater thermal plasticity induced by the acclimation to elevated temperatures and by heat-hardening in *Trichogramma* wasps [31], mites [32] and in other organisms, such as in the aquatic hydra/algae holobiont system [33].

Furthermore, a significantly higher gene abundance of bacterial symbionts, particularly of *B. aphidicola*, *S. symbiotic*, *R. insecticola* and *Rickettsia* spp., were recorded in the cohorts of temperature-tolerant aphid individuals (i.e., of mortality time periods 3 and 4) as compared to susceptible ones (i.e., of mortality time periods 1 and 2) for both aphid species. Likewise, the gene abundance of *B. aphidicola*, *S. symbiotica* and *H. defensa* were significantly higher in the acclimated than non-acclimated (basal) aphid individuals of both aphid species and for *R. insecticola* for *R. padi*. More interestingly, a significant and positive correlation was found among the thermal tolerance indices and gene abundance of total symbionts density, *B. aphidicola*, *S. symbiotica*, *H. defensa*, *R. insecticola* and *Spiroplasma* spp. for *R. padi*, and with the total symbionts density, total bacteria (16S rRNA) and with all aphid-specific bacterial symbionts (except *Spiroplasma* spp.) for *S. avenae*.

These results validate the potential role of aphid symbiotic bacteria in conferring ecological fitness and thermal tolerance to their host aphids [24,25,26,28,34,35]. Insect–microbial symbiont interactions play a vital role in the evolutionary adaptation of host insects to ecological stresses, such as extreme thermal exposures [36]. Many previous studies have demonstrated the significance of endosymbionts *S. symbiotica* and *H. defensa* in improving aphid tolerance to extreme temperature exposures [26,34,35,36,37,38]. Russell and Moran [35], Montllor et al. [34] and Dunbar et al. [39] demonstrated that bacterial the symbionts *S. symbiotica, H. defensa* and *B. aphidicola* ameliorate the thermal tolerance of their hosts and confer tolerance to high temperature exposures. However, how short-term acclimation boosted the gene abundance in the individuals of both species compared to basal (non-acclimated) needs further investigation. In-large, the results of this study corroborate that the interactions of aphids and their bacterial symbionts may play an important role in aphids’ thermal adaptation to high temperature exposures or events.

## 5. Conclusions

In short, a significantly higher abundance of bacterial symbionts was harbored by the individuals of the *R. padi* and *S. avenae* species tolerant to chronic thermal exposures than the susceptible ones. Short-term acclimation to 34 °C considerably enhanced the CTmax (thermal tolerance) for both aphid species. Furthermore, interestingly, the critical thermal maxima values of both species were positively associated with the gene abundance of *B. aphidicola*, *S. symbiotica*, *H. defensa* and *R. insecticola* signifying their putative role in conferring thermal tolerance to their host aphids. In future studies, the diversity and community structure of bacterial symbionts associated with these aphids should be conducted by Illumina deep sequencing of 16S rRNA.

## Figures and Tables

**Table 1:** Primer sequences used for the PCR amplification of total and aphid-specific bacterial symbionts of cereal aphids.

Symbiont Category	Type	Taxonomic Name	Bacterial GROUP	Gene Length (kb)	Primer Code	Real Time Quantitative PCR Primer Sequence (5′ to 3′)
Total Bacterial Community (16S rRNA)	Eubacteria			1.47	16S rRNAF	CCTACGGGAGGCAGCAG
					16S rRNAR	ATTCCGCGGCTGGCA
Primary/Obligate Symbiont	P-type	*Buchnera aphidicola*	Gammaproteobacterim	1.5	BaF	TGAGAGGATAACCAGCCACAC
					BaR	ATTTTTTTCCTCCCCGCTGA
Secondary/Facultative Symbionts	R-type/PASS	*Serratia symbiotica*	Gammaproteobacterim	1.46	SsF	CCCTGGACAAAGACTGACGC
					SsR	CGACATCGTTTACAGCGTGGA
	T-type/PABS	*Hamiltonella defensa*	Gammaproteobacterim	1.3	HdF	CCTCCTAAACGAATACTGACGC
					HdR	CACTTCTCTTGGAAACAACCTC
	U-type/PAUS	*Regiella insecticola*	Gammaproteobacterim	1.3	RiF	AGCCACACTGGAACTGAGAAAC
					RiR	TCCTCCCCGCTGAAAGTGCT
	S-type/PAR	*Rickettsia* spp.	Alphaprotobacterium	1.46	RspF	AGTGGCGAAGGCTGTCATCT
					RspR	GCTGCGAAACTGAAAGAAAATC
		*Spiroplasma* spp.	Mollicutes	1.4	SspF	GCGGTAATACATAGGTGGCAAGC
					SspR	AAGGCGGTTAGGGGTTGAGC
					BaR	ATTTTTTTCCTCCCCGCTGA

**Table 2:** Correlation among the critical thermal maxima (CTmax) of cereal aphids with their bacterial symbiont gene abundance.

Bacterial Symbionts	*Rhopalosiphum padi*	*Sitobion avenae*
	(*n* = 99)	(*n* = 99)
Total eubacterial community	0.298	0.670 **
*Buchnera aphidicola*	0.529 **	0.939 **
*Serratia symbiotica*	0.481 **	0.850 **
*Hamiltonella defensa*	0.433 *	0.658 **
*Regiella insecticola*	0.414 *	0.618 **
*Rickettsia* spp.	0.248	0.390 *
*Spiroplasma* spp.	0.473 **	0.048
Total symbionts density	0.514 **	0.350 *

Spearman’s Rank Correlation Coefficients (rho); * = correlation is significant at the 0.05 level (2-tailed); ** = correlation is significant at the 0.01 level (2-tailed).

## Data Availability

The data presented in this study are available in article.

## Supplementary Materials

The following are available online at https://www.mdpi.com/article/10.3390/insects13030231/s1, Figure S1: Effect of different acclimation times on critical thermal maxima (CTmax) of 5-day-old apterous adults of cereal aphid *Sitobion avenae*; Figure S2: Cumulative percent mortality of cereal aphids *Rhopalosiphum padi* and *Sitobion avenae* under 31 °C for different exposure times; Figure S3: Gene copy numbers (mean ± SD) of total (16S rRNA) and aphid-specific bacterial symbionts in basal and acclimated 5-day-old apterous adults of cereal aphids *Rhopalosiphum padi* (RP) and *Sitobion avenae* (SA).

## References

[1] Ma C.-S., Ma G., Pincebourde S. (2021). Survive a Warming Climate: Insect Responses to Extreme High Temperatures. Annu. Rev. Èntomol..

[2] Harvey J.A., Heinen R., Gols R., Thakur M.P. (2020). Climate change-mediated temperature extremes and insects: From outbreaks to breakdowns. Glob. Chang. Biol..

[3] Ma G., Rudolf V.H.W., Ma C. (2014). Extreme temperature events alter demographic rates, relative fitness, and community structure. Glob. Chang. Biol..

[4] Deutsch C.A., Tewksbury J.J., Huey R., Sheldon K.S., Ghalambor C., Haak D., Martin P.R. (2008). Impacts of climate warming on terrestrial ectotherms across latitude. Proc. Natl. Acad. Sci. USA.

[5] Everatt M., Convey P., Worland M., Bale J., Hayward S. (2013). Heat tolerance and physiological plasticity in the Antarctic collembolan, Cryptopygus antarcticus, and mite, Alaskozetes antarcticus. J. Therm. Biol..

[6] Somero G.N. (2005). Linking biogeography to physiology: Evolutionary and acclimatory adjustments of thermal limits. Front. Zool..

[7] Hofstetter R.W., Dempsey T.D., Klepzig K.D., Ayres M.P. (2007). Temperature-dependent effects on mutualistic, antagonistic, and commensalistic interactions among insects, fungi and mites. Community Ecol..

[8] Musolin D.L. (2007). Insects in a warmer world: Ecological, physiological and life-history responses of true bugs (Heteroptera) to climate change. Glob. Change Biol..

[9] Auad A.M., Alves S.O., Antunes-Carvalho C., Silva D.M., Resende T.T., Veríssimo B.A. (2009). The Impact of Temperature on Biological Aspects and Life Table ofRhopalosiphum padi(Hemiptera: Aphididae) Fed with Signal Grass. Fla. Èntomol..

[10] Ma G., Ma C.-S. (2012). Effect of acclimation on heat-escape temperatures of two aphid species: Implications for estimating behavioral response of insects to climate warming. J. Insect Physiol..

[11] Ma G., Hoffmann A., Ma C.-S. (2015). Daily temperature extremes play an important role in predicting thermal effects. J. Exp. Biol..

[12] Ma G., Bai C.-M., Wang X.-J., Majeed M.Z., Ma C.-S. (2018). Behavioural thermoregulation alters microhabitat utilization and demographic rates in ectothermic invertebrates. Anim. Behav..

[13] Zhu L., Hoffmann A.A., Li S., Ma C. (2021). Extreme climate shifts pest dominance hierarchy through thermal evolution and transgenerational plasticity. Funct. Ecol..

[14] Tsuchida T., Koga R., Shibao H., Matsumoto T., Fukatsu T. (2002). Diversity and geographic distribution of secondary endosymbiotic bacteria in natural populations of the pea aphid, Acyrthosiphon pisum. Mol. Ecol..

[15] Moran N.A., Russell J.A., Koga R., Fukatsu T. (2005). Evolutionary Relationships of Three New Species of Enterobacteriaceae Living as Symbionts of Aphids and Other Insects. Appl. Environ. Microbiol..

[16] Sakurai M., Koga R., Tsuchida T., Meng X.Y., Fukatsu T. (2005). Rickettsia symbiont in the pea aphid Acyrthosiphon pisum: Novel cellular tropism, effect on host fitness, and interaction with the essential symbiont Buchnera. Appl. Environ. Microbiol..

[17] Zytynska S.E., Weisser W. (2015). The natural occurrence of secondary bacterial symbionts in aphids. Ecol. Èntomol..

[18] Sepúlveda D.A., Zepeda-Paulo F., Ramírez C.C., Lavandero B., Figueroa C.C. (2017). Diversity, frequency, and geographic distribution of facultative bacterial endosymbionts in introduced aphid pests. Insect Sci..

[19] Douglas A.E. (1998). Nutritional Interactions in Insect-Microbial Symbioses: Aphids and Their Symbiotic Bacteria Buchnera. Annu. Rev. Èntomol..

[20] Moran N.A., Plague G.R., Sandström J.P., Wilcox J.L. (2003). A genomic perspective on nutrient provisioning by bacterial symbionts of insects. Proc. Nat. Acad. Sci. USA.

[21] Hansen A.K., Moran N.A. (2013). The impact of microbial symbionts on host plant utilization by herbivorous insects. Mol. Ecol..

[22] Douglas A.E. (2015). Multiorganismal Insects: Diversity and Function of Resident Microorganisms. Annu. Rev. Èntomol..

[23] Giron D., Dedeine F., Dubreuil G., Huguet E., Mouton L., Outreman Y., Vavre F., Simon J.-C. (2017). Influence of Microbial Symbionts on Plant–Insect Interactions. Adv. Bot. Res..

[24] Sabater-Muñoz B., Toft C. (2020). Evolution from Free-Living Bacteria to Endosymbionts of Insects: Genomic Changes and the Importance of the Chaperonin GroEL. Symbiosis: Cellular, Molecular, Medical and Evolutionary Aspects.

[25] Burke G.R., McLaughlin H.J., Simon J.C., Moran N.A. (2010). Dynamics of a re-current Buchnera mutation that affects thermal tolerance of pea aphid hosts. Genetics.

[26] Castañeda L.E., Sandrock C., Vorburger C. (2010). Variation and covariation of life history traits in aphids are related to infection with the facultative bacterial endosymbiont Hamiltonella defensa. Biol. J. Linn. Soc..

[27] Mandrioli M., Manicardi G.C. (2013). Evolving aphids: One genome-one organism insects or holobionts?. Invert. Surv. J..

[28] Zhang B., Leonard S.P., Li Y., Moran N.A. (2019). Obligate bacterial endosymbionts limit thermal tolerance of insect host species. Proc. Natl. Acad. Sci. USA.

[29] Cao J.-Y., Xing K., Liu H.-P., Zhao F. (2018). Effects of developmental acclimation on fitness costs differ between two aphid species. J. Therm. Biol..

[30] Turak E., Talent R., Sunnucks P., Hales D.F. (1998). Different responses to temperature in three closely-related sympatric cereal aphids. Èntomol. Exp. Appl..

[31] Scott M., Berrigan D., Hoffmann A. (1997). Costs and benefits of acclimation to elevated temperature in Trichogramma carverae. Èntomol. Exp. Appl..

[32] Hart A., Bale J., Tullett A., Worland M., Walters K. (2002). Effects of temperature on the establishment potential of the predatory mite Amblyseius californicus McGregor (Acari: Phytoseiidae) in the UK. J. Insect Physiol..

[33] Ye S., Badhiwala K.N., Robinson J.T., Cho W.H., Siemann E. (2019). Thermal plasticity of a freshwater cnidarian holobiont: Detection of trans-generational effects in asexually reproducing hosts and symbionts. ISME J..

[34] Montllor C.B., Maxmen A., Purcell A.H. (2002). Facultative bacterial endosymbionts benefit pea aphids Acyrthosiphon pisum under heat stress. Ecol. Entomol..

[35] Russell J.A., Moran N.A. (2005). Costs and benefits of symbiont infection in aphids: Variation among symbionts and across temperatures. Proc. R. Soc. B Boil. Sci..

[36] Zhang Q.-G., Buckling A. (2011). Antagonistic coevolution limits population persistence of a virus in a thermally deteriorating environment. Ecol. Lett..

[37] Chen D.-Q., Montllor C.B., Purcell A.H. (2000). Fitness effects of two facultative endosymbiotic bacteria on the pea aphid, Acyrthosiphon pisum, and the blue alfalfa aphid, A. kondoi. Èntomol. Exp. Appl..

[38] Oliver K.M., Campos J., Moran N.A., Hunter M.S. (2007). Population dynamics of defensive symbionts in aphids. Proc. Biol. Sci..

[39] Dunbar H.E., Wilson A.C.C., Ferguson N.R., Moran N.A. (2007). Aphid Thermal Tolerance Is Governed by a Point Mutation in Bacterial Symbionts. PLoS Biol..

