# Supplementary material for: Bacterial Symbionts Confer Thermal Tolerance to Cereal Aphids Rhopalosiphum padi and Sitobion avenae
Source: Insects. 2022 Feb 25;13(3):231. doi: 10.3390/insects13030231 (PMC8949882; doi:10.3390/insects13030231)
Supplement: Supplementary file 1 [file insects-13-00231-s001.zip › insects-1548893-supplementary.pdf]

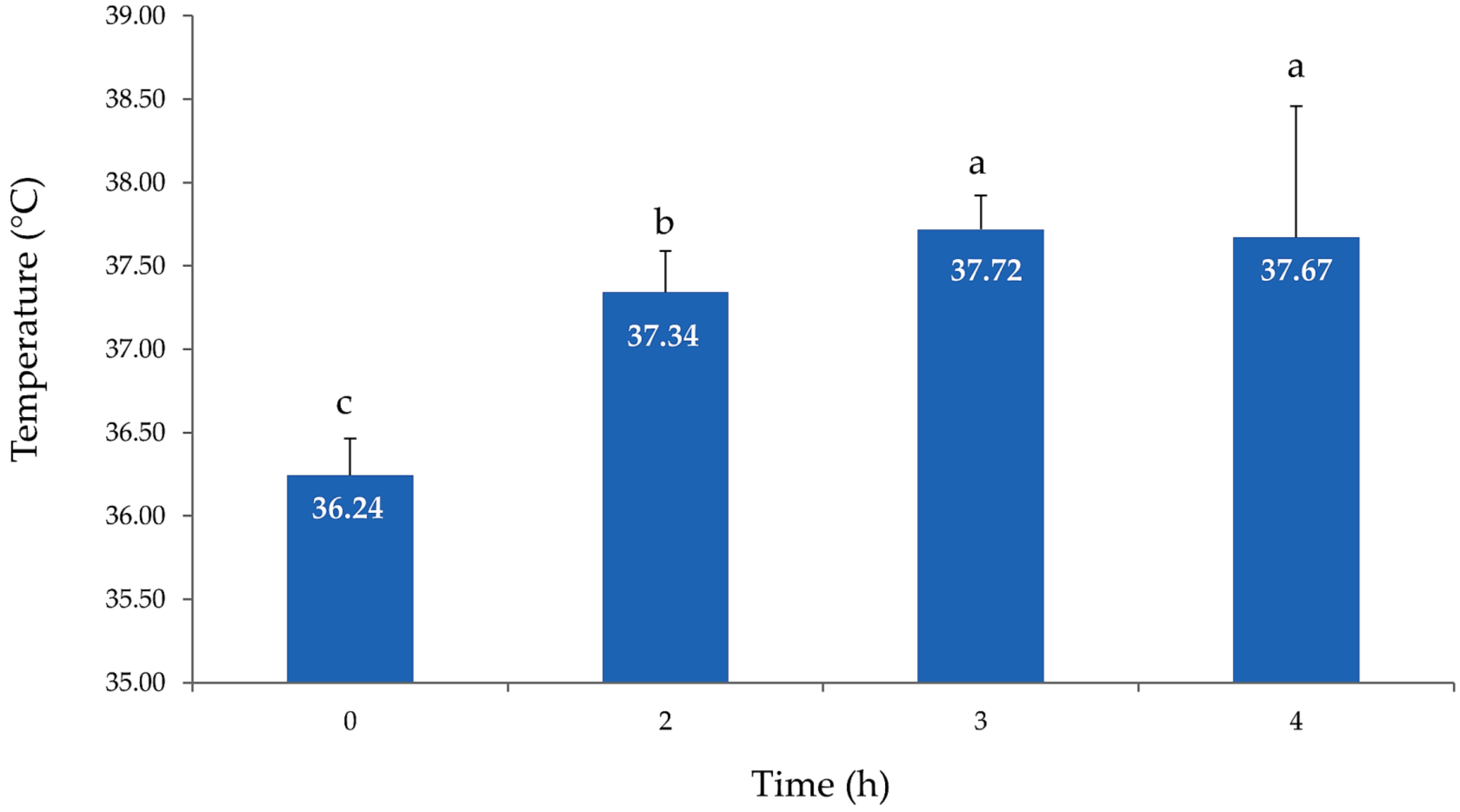

Figure S1. Effect of different acclimation times on critical thermal maxima (CTmax) of 5-day old apterous adults of cereal aphid *Sitobion avenae*. Values are means (± SD) of 33 independent observations for each treatment. Different letters above columns represent statistical significance among the treatments (one-way ANOVA; $p < 0.01$).

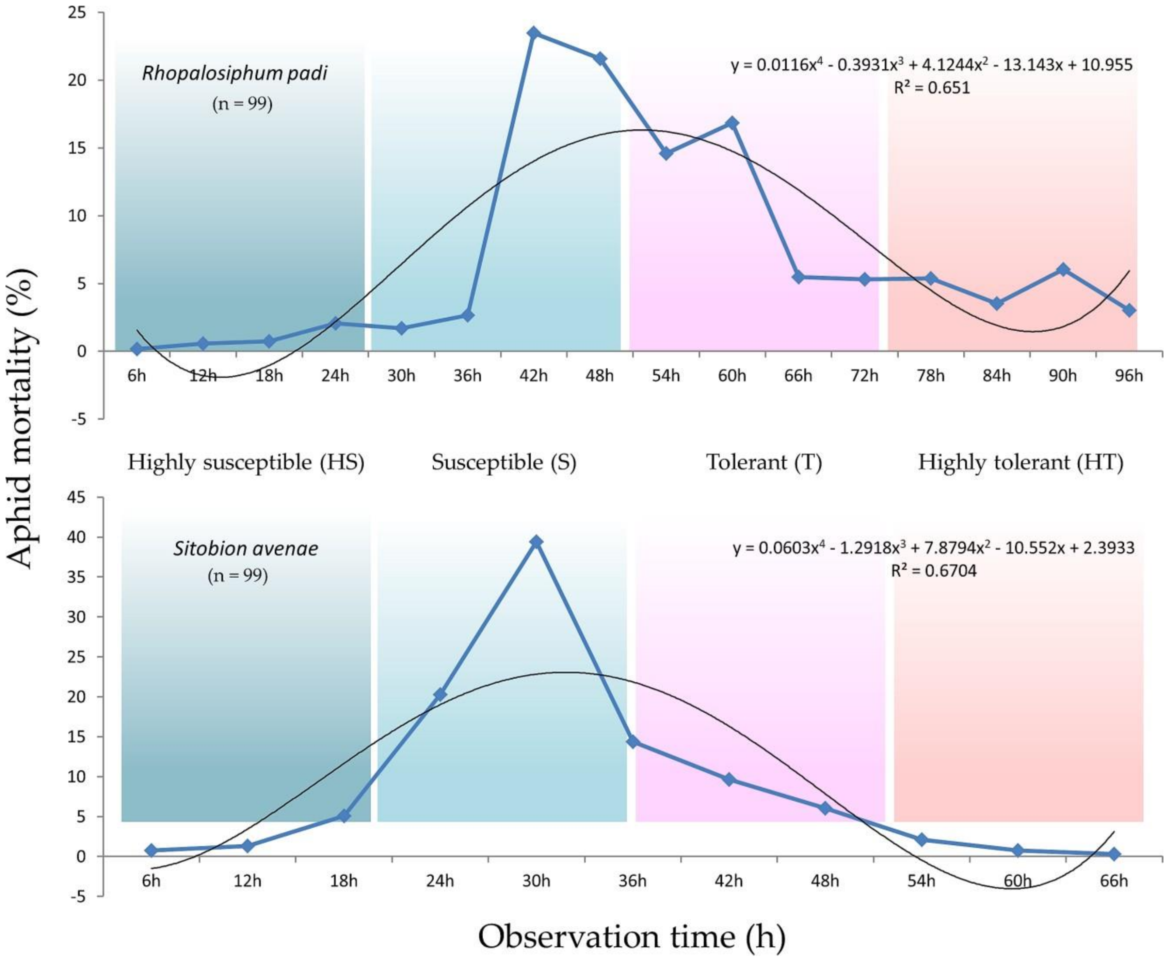

Figure S2. Cumulative percent mortality of cereal aphids *Rhopalosiphum padi* and *Sitobion avenae* under 31 °C for different exposure times. For each species, 33 aphid individuals were exposed from each of the laboratory reared F_1, F_2 and F_3 generations. Best-fit polynomial (order 4) response curves are shown along with the regression equations and R^2 values.

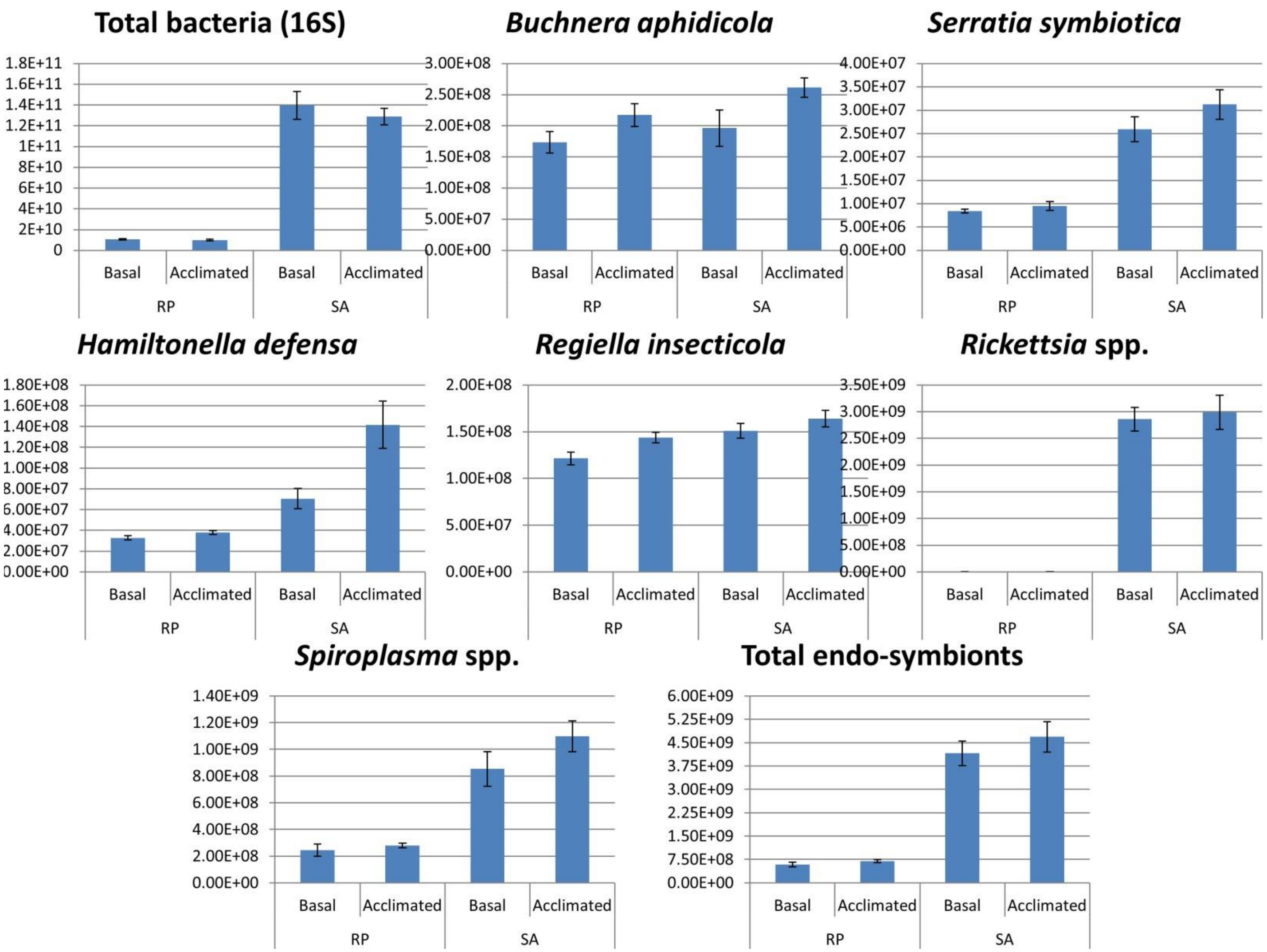

Figure S3. Gene copy numbers (mean ± SD) of total (16S rRNA) and aphid-specific bacterial symbionts in basal and acclimated 5-day old apterous adults of cereal aphids *Rhopalosiphum padi* (RP) and *Sitobion avenae* (SA).
